# Corticosteroid-Induced Liver Injury in Adult-Onset Still’s Disease

**DOI:** 10.3390/medicina58020191

**Published:** 2022-01-27

**Authors:** Chin-Chi Lee, Yi-Jen Peng, Chun-Chi Lu, Hsiang-Cheng Chen, Fu-Chiang Yeh

**Affiliations:** 1 Department of Internal Medicine, Tri-Service General Hospital, National Defense Medical Center, Taipei 11490, Taiwan; chanbers0@gmail.com (C.-C.L.); jameslutaiwan@gmail.com (C.-C.L.); hccheng@mail.ndmctsgh.edu.tw (H.-C.C.); 2 Department of Pathology, Tri-Service General Hospital, National Defense Medical Center, Taipei 11490, Taiwan; yijen0426@gmail.com; 3 Division of Rheumatology, Immunology and Allergy, Department of Internal Medicine, Tri-Service General Hospital, National Defense Medical Center, Taipei 11490, Taiwan; 4 Division of Rheumatology, Immunology and Allergy, Department of Internal Medicine, Penghu Branch, Tri-Service General Hospital, Penghu 880, Taiwan

**Keywords:** adult-onset Still’s disease, corticosteroids, drug-induced liver injury

## Abstract

*Background and Objectives*: Adult-onset Still’s disease (AOSD) is a rheumatic disease characterized by systemic inflammatory symptoms, including intermittent spiking fever, polyarthritis and a distinctive salmon-colored rash. Corticosteroids are the first-line treatment for AOSD. However, corticosteroids are potentially hepatotoxic in certain cases and may complicate the course of the disease. *Materials and Methods*: A 29-year-old female suffering from fever of unknown origin for two weeks was diagnosed with AOSD according to Yamaguchi’s criteria. She received corticosteroids as the first-line treatment for AOSD and developed acute severe hepatitis. A diagnostic protocol has been performed. *Results*: Corticosteroid-induced liver injury was confirmed by clinical observation and rechallenge of the drug in this case. The result of liver biopsy also supported the diagnosis. Mycophenolic acid, a disease-modifying antirheumatic drug (DMARD) was chosen as an alternative treatment. AOSD remission was achieved under this treatment after three months. *Conclusions*: Severe acute hepatitis induced by corticosteroids, although very rare, may be observed in patients with AOSD. Drug-induced liver injury needs to be kept in mind when unexpected acute hepatitis is found. Mycophenolic acid could be a proper substitute medication in these cases.

## 1. Introduction

Adult-onset Still’s disease (AOSD) is a rheumatic disease presenting with systemic inflammatory symptoms, including spiking fever, polyarthritis and a typical salmon-colored rash, in addition to laboratory findings of leukocytosis, hyperferritinemia and abnormal liver function tests [1]. Corticosteroids with anti-inflammatory and immunomodulatory activities remain the first-line treatment for AOSD [2]. However, there is a rare risk of hepatotoxicity with corticosteroids, which may complicate the course of the disease and result in poor prognosis if unrecognized. Although corticosteroid-induced liver injury has been reported in several autoimmune diseases, the mechanism is still unclear [3]. Herein, to the best of our knowledge, we present the first case of corticosteroid-induced liver injury in a newly diagnosed AOSD.

## 2. Case Report

A 29-year-old Taiwanese female with a family history of Sjögren’s syndrome previously visited a district hospital due to two weeks of fever, arthralgia, sore throat and macular rashes. After a series of examinations over a period of one week, she was transferred to a tertiary hospital with a fever of unknown origin. On admission, repeated viral tests and bacterial cultures disclosed negative results. Pertinent laboratory findings showed leukocytosis (13,060/μL) with 85.5% of neutrophils and remarkable hyperferritinemia (12,585 ng/mL). Other biomarkers for rheumatic disease including antinuclear antibodies and rheumatoid factor were negative. Further Gallium-67 inflammation scans and computed tomography of the abdomen demonstrated the enlargement of multiple lymph nodes. Lymphoma was excluded by excisional lymph node biopsy. AOSD was finally diagnosed according to the Yamaguchi criteria [1].

**Figure 1:**
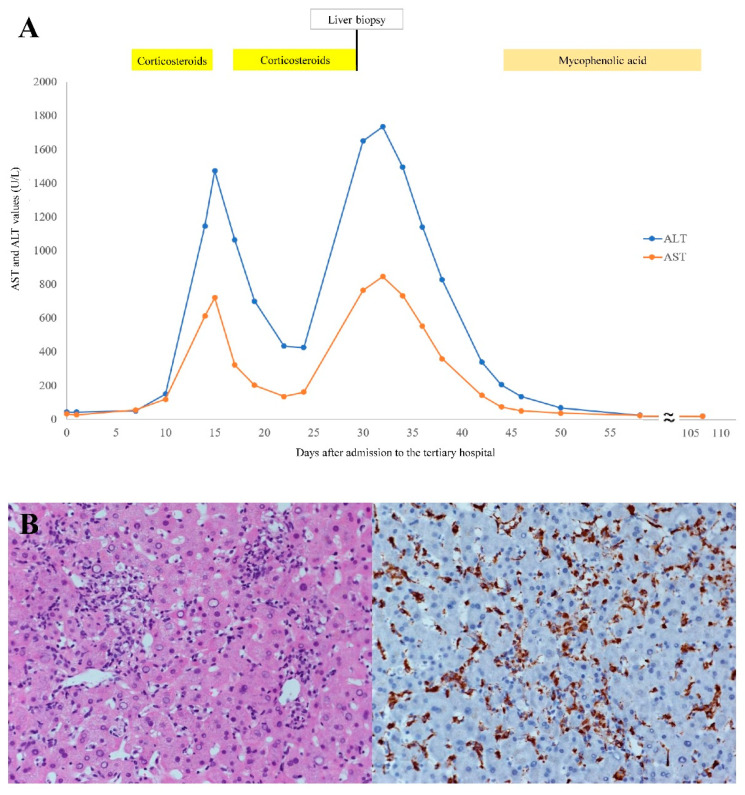
(**A**) Trend of liver enzymes since admission to the tertiary hospital; AST: aspartate aminotransferase; ALT: alanine aminotransferase. (**B**) Histopathology of the liver reveals (**left**) acute hepatitis with neutrophils and lymphocytes infiltration in H&E stain and (**right**) Kupffer cell proliferation highlighted by CD68 immunohistochemical stain (both 200X). H&E: hematoxylin and eosin.

Owing to a history of allergy to non-steroidal anti-inflammatory drugs (NSAIDs), intravenous betamethasone (8 mg/day) was initiated for treatment of AOSD on the 8th day and shifted to oral prednisolone (20 mg/day) two days later. Fever and arthralgia were resolved; however, elevated liver enzymes (alanine aminotransferase (ALT) 150 U/L, aspartate aminotransferase (AST) 119 U/L) were first noted on the 10th day and reached peak levels (ALT 1473 U/L, AST 722 U/L) on the 15th day. (Figure 1A) Under suspicion of drug-induced liver injury (DILI), all medications were stopped. In addition, viral and autoimmune hepatitis were both excluded again by serological tests. Fortunately, liver enzyme levels declined after drug cessation. Corticosteroids were resumed on the 17th day and the patient was discharged one week later when her liver function (ALT 427 U/L, AST 161 U/L) was much improved. (Figure 1A) Maintenance therapy for AOSD with prednisolone (20 mg/day) was prescribed.

Surprisingly, acute severe hepatitis (ALT 1652 U/L, AST 766 U/L) was found five days after discharge. The patient was re-admitted and prednisolone was halted immediately while other medications including silymarin and lactulose were maintained. (Figure 1A) Histopathology of the liver revealed acute hepatitis featuring infiltration of lymphocytes, neutrophils, and proliferative Kupffer cells as well as occasional apoptotic bodies in lobular and portal areas. DILI was considered after excluding viral and autoimmune liver diseases. (Figure 1B) Remission of hepatitis was observed after removal of prednisolone. Hence, corticosteroid-induced liver injury was confirmed based on the clinical findings and the positive prednisolone re-challenge. Mycophenolic acid was substituted for prednisolone for treatment of AOSD on account of its lower risk of hepatotoxicity [4]. No relapse of the disease or liver function impairment was noted in the following three months. (Figure 1A).

## 3. Discussion

Differential diagnosis of acute hepatitis in AOSD is challenging. The liver injury can arise from numerous causes, such as direct complication of the disease, AOSD-induced macrophage activation syndrome (MAS) and DILI [5,6]. A retrospective study of Chinese AOSD patients demonstrated abnormal liver enzymes in 62.3% of patients, among which many had slight to moderate hepatitis. Additionally, most of them achieved complete remission with systemic corticosteroids [5]. Moreover, young AOSD patients with cytopenia were more likely to suffer from acute severe hepatitis related to reactive MAS with high mortality [2]. Importantly, in the present case liver dysfunction was not observed until the administration of corticosteroids and spontaneously resolved after the removal of the medication. Therefore, corticosteroid-induced liver injury was impressed.

DILI is rarely reported in AOSD and has been observed in patients treated with NSAIDs, anakinra and tocilizumab. Corticosteroids, due to their potent immunomodulatory activity, are the rescue therapy for these cases [6,7,8]. Paradoxically, corticosteroid-related DILI has been increasingly reported recently in patients with autoimmune phenotypes, particularly multiple sclerosis and Graves’ disease. It typically emerges during the first weeks after exposure to high dose corticosteroids, presenting with hepatocellular damage, and usually goes unnoticed favoring rechallenge [3]. Unexpectedly, the relatively low dose prednisolone (20 mg/day) in this case induced acute severe hepatitis and was confirmed by positive rechallenge of the drug.

The mechanism of corticosteroid-induced liver injury is still unknown. Low doses of corticosteroids are considered safe for hepatic function, but long-term use of the drug may result in steatohepatitis. High doses of corticosteroids can rarely cause intrinsic hepatotoxicity. Corticosteroids are metabolized by cytochrome P450 3A4 (CYP3A4). Metabolic idiosyncrasy resulting from aberrant hepatic metabolism should be considered the mechanism of corticosteroid-induced hepatotoxicity [9].

Given that NSAIDs and corticosteroids were contraindicated, second-line treatment with disease-modifying antirheumatic drugs (DMARDs) was indispensable. Although methotrexate is suggested as the first-line steroid-sparing therapy in AOSD, the high risk of hepatotoxicity limits its use in the present case. Mycophenolic acid was preferred over other DMARDs as a maintenance treatment because its efficacy and safety were reported in an AOSD patient intolerant to methotrexate [4].

Concerning AOSD pathogenesis, dysregulation of Th17 cells and elevated serum levels of interleukin 17 (IL-17) have been observed. The rationale for mycophenolic acid treatment in AOSD is that it has an impact on T cell response and a strong inhibitory effect on IL-17 production [4]. Although mycophenolic acid treatment in AOSD is only seen in case reports, it has been widely used in severe autoimmune/inflammatory disease such as lupus nephritis. In this case, mycophenolic acid treatment for AOSD was successful because the symptoms and signs of AOSD, such as fever, arthritis and hyperferritinemia, all subsided within three months.

## 4. Conclusions

In conclusion, corticosteroids, although uncommon, may cause severe hepatitis in AOSD. In these cases, mycophenolic acid could be an ideal alternative treatment.

## Data Availability

The data that support the findings of this study are available from the corresponding author, F.C.Y, upon reasonable request.
